# Clinical Judgment vs Triage Scales for Detecting Large Vessel Occlusions in Suspected Acute Stroke

**DOI:** 10.1001/jamanetworkopen.2023.32894

**Published:** 2023-09-12

**Authors:** Eckhard Schlemm, Marius Piepke, Simon S. Kessner, Lukas Meyer, Bastian Cheng, Christian Gerloff, Götz Thomalla

**Affiliations:** 1 Department of Neurology, University Medical Center Hamburg-Eppendorf, Hamburg, Germany; 2 Department of Psychosomatic Medicine und Psychotherapy, University Medical Center Hamburg-Eppendorf, Hamburg, Germany; 3 Department of Diagnostic and Interventional Neuroradiology, University Medical Center Hamburg-Eppendorf, Hamburg, Germany

## Abstract

This cohort study examines clinical judgment of large vessel occlusions compared with triage scales in a sample of patients admitted to the emergency department with suspicion of acute stroke.

## Introduction

Endovascular therapy is effective for acute stroke with large vessel occlusion (LVO). To avoid treatment delays, triage scales were designed for use by paramedics to identify patients for direct transport to endovascular-capable stroke centers.^1^ With telemedicine, clinicians’ judgments are becoming available in the prehospital setting,^2^ but their diagnostic accuracy is unknown.

## Methods

This cohort study was approved by the ethics review board of the Ärztekammer Hamburg, and informed consent was waived because patient data were deidentified. The study follows the Standards for Reporting of Diagnostic Accuracy (STARD) reporting guideline. Additional information regarding our methods and the study flow diagram can be found in the eMethods in Supplement 1.

We prospectively evaluated physicians’ assessment of LVO, hypothesizing that clinical judgment (CLUE) would be noninferior to common triage scales. The primary end point was the area under the receiver operating curve (AUROC) relative to acute vessel imaging.

Consecutive patients presenting to the emergency department (ED) with suspicion of acute stroke were identified between March and December 2022. Prior to cerebral imaging, treating physicians estimated the likelihood of an occlusion of a proximal intracranial artery on an analog scale from 0 to 100. Rapid Arterial Occlusion Evaluation (RACE) and Field Assessment Stroke Triage for Emergency Destination (FAST-ED) scores were computed from the National Institutes of Health stroke scale (NIHSS).

Point estimates and 95% CIs were obtained from multireader multicase regressions using R version 4.2 (R Project for Statistical Computing).^3^ Analyses were carried out in the intention-to-analyze population of patients receiving CLUE prior to vessel imaging and in the target population of patients with acute ischemic stroke or transient ischemic attack. Subgroup analyses explored the effects of age, sex, symptom severity, and symptom onset-to-door times. Statistical significance was set at *P* < .05, and tests were 2-sided. Data were analyzed from January to February 2023.

## Results

This study included 183 patients (95 [52%] women; median [IQR] age, 79 [67.3-85.6] years) (Table), and 46 (25%) had at least 1 LVO. Of a total of 49 LVOs, 32 occlusions (65%) affected the middle cerebral, 11 (22%) the intracranial internal carotid, 2 (4%) each the anterior and posterior cerebral, and 2 (4%) the basilar artery. Of the 36 physicians who assessed patients, 32 (89%) were residents with a median (IQR) 4 (1-6) years of postgraduate experience, and 4 (11%) were board-certified neurologists.

**Table.** Baseline Demographic and Clinical Variables and Distribution of CLUE, RACE, and FAST-ED Scores Stratified by Discharge Diagnosis

Variables	Discharge diagnosis, median (IQR)				
	All	LVO	AIS without LVO, or TIA	ICH	Stroke mimic
Patients, No. (%)	183 (100)	46 (25.1)	79 (43.2)	13 (7.1)	45 (24.6)
Age, y	78.8 (67.3-85.6)	77.9 (64.3-84.2)	78.4 (67.8-85.6)	79.0 (68.1-84.0)	82.2 (70.5-86.9)
Sex, No. (%)					
Male	88 (48.1)	23 (50.0)	28 (35.4)	8 (61.5)	29 (64.4)
Female	95 (51.9)	23 (50.0)	51 (64.6)	5 (38.5)	16 (35.6)
NIHSS	6 (3-13)	13.5 (8-18.75)	4 (2.5-8)	22 (9-26)	4 (2-8)
IVT, No. (%)	47 (25.7)	27 (58.7)	16 (20.3)	0	0
DTN, min	31 (25-44.5)	30 (25.5-35.5)	34 (24-49.5)	NA	NA
EVT, No. (%)	34 (18.6)	34 (73.9)	0	0	0
DTG, min	83.5 (64.75-97.75)	83.5 (64.75-96.75)	NA	NA	NA
Scales, score					
CLUE, (0-100)^a^	20 (5-60)	80 (50-90)	10 (3.5-27.5)	20 (10-40)	5 (0-10)
RACE, (0-9)^a^	2 (0-8)	6 (3-8)	1 (0-3)	6 (3-6)	1 (0-3)
FAST-ED, (0-9)^a^	2 (1-4)	4 (3-6)	1 (0-2)	4 (2-6)	1 (0-3)

Abbreviations: AIS, acute ischemic stroke; CLUE, clinical judgment; DTG, door-to-groin time; DTN, door-to-needle time; EVT, endovascular therapy; FAST-ED, Field Assessment Stroke Triage for Emergency Destination; ICH, intracerebral hemorrhage; IVT, intravenous thrombolysis; LVO, large vessel occlusion; NA, not applicable; NIHSS, National Institutes of Health stroke scale; RACE, Rapid Arterial Occlusion Evaluation; TIA, transient ischemic attack.

^a^ For LVO-detection scales, ranges of possible scores for each scale are given in parentheses in the first column. Higher values represent more severe deficits.

The accuracy of CLUE to detect LVO was 0.96 (95% CI, 0.92 to 1.00) compared with RACE (0.79; 95% CI, 0.67 to 0.91), FAST-ED (0.76; 95% CI, 0.62 to 0.89), and NIHSS (0.70; 95% CI, 0.53 to 0.87). The difference between CLUE and RACE was 0.17 (95% CI, 0.07 to 0.26; *P* for noninferiority < .001; *P* for superiority = .001). The difference between CLUE and FAST-ED was 0.20 (0.11 to 0.30; *P* for noninferiority < .001; *P* for superiority < .001), between CLUE and NIHSS 0.26 (95% CI, 0.16 to 0.35). NIHSS was similar to RACE (difference in AUC, 0.09; 95% CI, 0 to 0.19) and FAST-ED (difference in AUC, 0.06; 95% CI, −0.04 to 0.15).

Subgroup analyses showed no heterogeneity with respect to age (<75 years vs ≥75 years), sex (male vs female), onset-to-door time (<6h vs ≥6h), or NIHSS (<10 vs ≥10) (Figure). Clinician-assessed likelihood of an LVO of at least 50% was more sensitive (0.85; 95% CI, 0.72-0.98) than a RACE score of at least 5 (0.63; 95% CI, 0.51-0.74), a FAST-ED score of at least 4 (0.65; 95% CI, 0.47-.82), and a NIHSS score of at least 10 (0.63; 95% CI, 0.48-0.79). With the same cut-offs, clinical judgment was more specific (0.88; 95% CI, 0.71-1.00) than FAST-ED (0.75; 95% CI, 0.59-0.92) and NIHSS (0.70; 95% CI, 0.49-0.92), with a similar specificity to RACE (0.79; 95% CI, 0.57-1.00).

**Figure.**
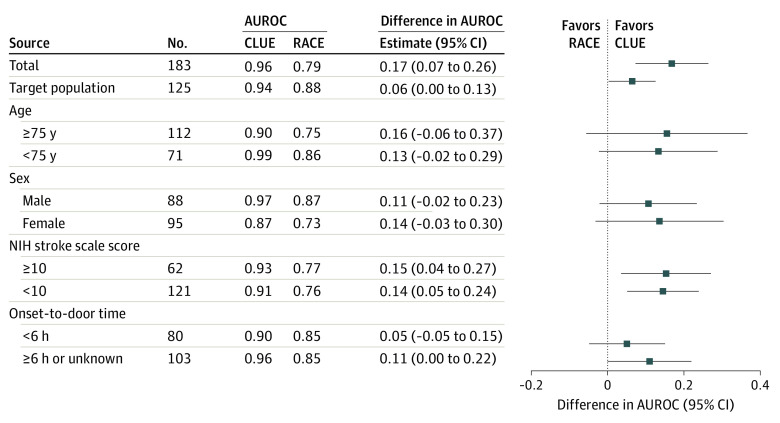
Diagnostic Accuracy of Clinical Judgment (CLUE) and Rapid Arterial Occlusion Evaluation (RACE) Scale Comparison of the accuracies of CLUE and RACE scale for detecting acute large vessel occlusion in relation to noninvasive intracranial vessel imaging measured by the area under the receiver operating curve (AUROC) in the whole study population (n = 183); the target population of patients with ischemic stroke or transient ischemic attack (n = 125); and stratified by prespecified subgroups. Boxes represent estimates of differences in AUROCs, and line segments represent 95% CIs.

## Discussion

The findings of this cohort study suggest that CLUE is not inferior, and in fact superior, to LVO triage scales. In patients with ischemic stroke or TIA, the diagnostic gain was smaller than in the full cohort due to a lower accuracy of CLUE and higher accuracy of RACE. This suggests benefits of clinical expertise in differentiating stroke from mimics and identifying LVO in patients with confirmed cerebral ischemia. CLUE performed well in subgroups associated with diagnostic bias or differential eligibility for EVT.

Clinical trials investigating LVO-detection scales have yielded mixed results. While in observational studies scale-based triage improved access to thrombectomy,^4,5^ bypassing the closest stroke-ready hospital in a rural region was not associated with clinical benefit.^6^ Limitations of our study include possible selection bias toward patients with more severe symptoms, assessment of patients in the ED, and judgment by neurology residents.

Further research is needed to validate our findings in independent cohorts and prehospital settings. Our findings, if replicated, suggest the possibility of extended telemedicine approaches that integrate emergency medical services with stroke hospitals, make clinical expertise available in the ambulance, and allow remote CLUE-based triaging of patients with suspected LVO.

## Notes

Supplement 1.
eMethods.

Supplement 2.
Data Sharing Statement
