# Supplementary material for: Clinical Judgment vs Triage Scales for Detecting Large Vessel Occlusions in Suspected Acute Stroke
Source: JAMA Netw Open. 2023 Sep 12;6(9):e2332894. doi: 10.1001/jamanetworkopen.2023.32894 (PMC10498329; doi:10.1001/jamanetworkopen.2023.32894)
Supplement: Supplement 1. — eMethods. [file jamanetwopen-e2332894-s001.pdf]

Supplemental Online Content

Schlemm E, Piepke M, Kessner SS, et al. Clinical judgment vs triage scales for predicting large vessel occlusions in suspected acute stroke. *JAMA Netw Open*. 2023;6(9):e2332894. doi:10.1001/jamanetworkopen.2023.32894

eMethods.

This supplemental material has been provided by the authors to give readers additional information about their work.

© 2023 Schlemm E et al. *JAMA Network Open*.

eMethods.

Test methods

For the index test, the treating stroke physicians were asked to estimate and document, after clinical evaluation and prior to any cerebral imaging, the likelihood that an occlusion of a proximal intracranial artery (intracranial internal carotid; first or second segment of the anterior, middle or posterior cerebral; or basilar artery) was causing the patient's presenting symptoms. This index test was scored on an analog scale from 0 to 100, representing the estimated probability, or relative frequency, with which a hypothetical patient, similar to the presenting subject in all known regards, would harbor an LVO. Assessors were encouraged to consider not only their neurological examination, but also the patient's medical history including comorbidities and prescribed medication as relayed by the patient or EMS personnel, as well as vital parameters and other clinical findings. In addition, assessors performed and documented the results of an NIHSS examination, from which the RACE and Field Assessment Stroke Triage for Emergency Destination (FAST-ED)[1] scales as comparator tests were computed. On both scales, possible values range from 0 to 9, with higher values indicating more severe neurological deficits. Acute cerebral vessel imaging by either CT angiography or MRI was performed as clinically indicated at the discretion of the treating physician. Information on patency of intracranial arteries was extracted from the final radiology report; this reference test was considered positive if a proximal vessel occlusion consistent with the patient's acute symptoms and without clear signs of chronicity was described. By design, reference test results were not available to performers of the index test at the time of assessment. Reporting neuroradiologists were agnostic to the results of the index test, but not to routine clinical information.

No indeterminate results were possible on the index test. Indeterminate results on the reference test were considered positive if they led to endovascular treatment, and negative otherwise.

© 2023 Schlemm E et al. *JAMA Network Open*.

Multi-reader multi-case modelling

Patients were included in the analysis if they received acute intracranial vessel imaging and CLUE was performed prior to imaging. Point estimates and 95% confidence intervals for the individual AUCs as well as their differences were obtained from the linear mixed-effects representation of a special case of the multi-reader multi-case (MRMC) model in which case is fully nested within reader to account for dependencies between assessments by the same reader.[2,3] Thus, the empirical AUC of test *i* performed by rater *j* on multiple cases was modeled as $\mathrm{AUC}_{i,j} = \mu + \tau_i + R_j + +(\tau R)_{i,j} + \varepsilon_{i,j}$, where μ denotes an overall mean, and τ_i, R_j, $(\tau R)_{i,j}$ denote a fixed test effect, a random rater effect, and a random test × rater interaction effect, respectively. The random terms R_j, $(\tau R)_{i,j}$, and the random error $\epsilon_{i,j}$ were assumed to be independent and normally distributed with mean zero.

Both case and rater were modeled as random factors in order to ensure generalizability of our results to unseen patients and physicians, and to account for variability in diagnostic accuracy between raters. Raters who had assessed fewer than two patients each with and without LVO were pooled into a single common category to allow estimation of within-factor variance.

Sample size estimation

Sample size determination was based on an AUC of the RACE scale of 80%,[4,5] an LVO prevalence in the target population of patients with cerebral ischemia (AIS or TIA) of 20%,[6] the non-inferiority margin of -0.05, a significance level of 5% and a power of 80%. Simulations using the Roe—Metz—Hillis model[7] with default covariance parameters[8] indicated that with an anticipated number of ten readers, 300 cases including 50 LVO patients would be sufficient to achieve the desired power. Based on retrospective observational data, we expected 50 `code stroke` patients at our ED per month, of whom 30 would receive cerebral vessel imaging and five would have an LVO. Hence, a recruitment period of ten months was determined.

© 2023 Schlemm E et al. *JAMA Network Open*.

Hypothesis testing

The primary endpoint of the study was the area under the receiver-operating curve (AUROC) as a measure of the diagnostic accuracies of both the clinicians' subjective assessment of the likelihood of an LVO (the index test) and two NIHSS-derived LVO-detection scales (RACE, FAST-ED; the comparators) relative to the gold standard of non-invasive acute vessel imaging (the reference test). The non-inferiority margin for the main hypothesis was set to 0.05 and, consequently, clinical judgment was considered non-inferior to LVO-detection scales if the lower boundary of the 95%-confidence intervals for the difference between the AUCs of CLUE and RACE and the difference between the AUCs of CLUE and FAST-ED both exceeded -0.05. Since type I error rates under this procedure are not inflated,[9] no correction for multiple comparisons was applied.

Study Flow Diagram

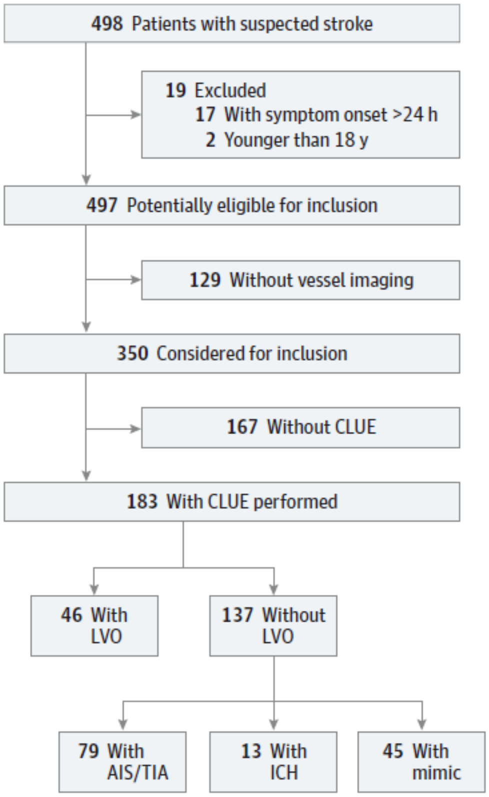

© 2023 Schlemm E et al. *JAMA Network Open*.

Supplemental references

1. Lima FO, Silva GS, Furie KL, Frankel MR, Lev MH, Camargo ÉCS, Haussen DC, Singhal AB, Koroshetz WJ, Smith WS, et al. Field Assessment Stroke Triage for Emergency Destination: A Simple and Accurate Prehospital Scale to Detect Large Vessel Occlusion Strokes. *Stroke*. 2016;47:1997–2002.
2. Hillis SL, Obuchowski NA, Schartz KM, Berbaum KS. A comparison of the Dorfman-Berbaum-Metz and Obuchowski-Rockette methods for receiver operating characteristic (ROC) data. *Stat. Med.* 2005;24:1579–1607.
3. Hillis SL. Relationship between Roe and Metz simulation model for multireader diagnostic data and Obuchowski-Rockette model parameters. *Stat. Med.* 2018;37:2067–2093.
4. Duvekot MHC, Venema E, Rozeman AD, Moudrous W, Vermeij FH, Biekart M, Lingsma HF, Maasland L, Wijnhoud AD, Mulder LJMM, et al. Comparison of eight prehospital stroke scales to detect intracranial large-vessel occlusion in suspected stroke (PRESTO): a prospective observational study. *Lancet Neurol.* 2021;20:213–221.
5. Nguyen TTM, Van Den Wijngaard IR, Bosch J, Van Belle E, Van Zwet EW, Dofferhoff-Vermeulen T, Duijndam D, Koster GT, De Schryver ELLM, Kloos LMH, et al. Comparison of Prehospital Scales for Predicting Large Anterior Vessel Occlusion in the Ambulance Setting. *JAMA Neurol.* 2021;78:157–164.
6. Sporns PB, Brehm A, Hilgers C, Ntoulias N, Tsogkas I, Psychogios M. Distribution of Diagnoses and Clinical and Imaging Characteristics in 1,322 Consecutive Suspected Stroke Patients. *Front. Neurol.* 2021;12:1822.
7. Hillis SL, Obuchowski NA, Berbaum KS. Power Estimation for Multireader ROC Methods. An Updated and Unified Approach. *Acad. Radiol.* 2011;18:129–142.
8. Roe CA, Metz CE. Dorfman-Berbaum-Metz method for statistical analysis of multireader, multimodality receiver operating characteristic data: Validation with computer simulation. *Acad. Radiol.* 1997;4:298–303.
9. U.S. FDA. Guidance for Industry: Multiple Endpoints in Clinical Trials [Internet]. 2022;

© 2023 Schlemm E et al. *JAMA Network Open*.
