# Supplementary material for: Clinical Judgment vs Triage Scales for Detecting Large Vessel Occlusions in Suspected Acute Stroke
Source: JAMA Netw Open. 2023 Sep 12;6(9):e2332894. doi: 10.1001/jamanetworkopen.2023.32894 (PMC10498329; doi:10.1001/jamanetworkopen.2023.32894)
Supplement: Supplement 2. — Data Sharing Statement [file jamanetwopen-e2332894-s002.pdf]

Data Sharing Statement

Data
Data available: Yes
Data types: Deidentified participant data, Data dictionary
How to access data: e.schlemm@uke.de
When available: With publication

Supporting Documents
Document types: Statistical/analytic code
How to access documents: e.schlemm@uke.de
When available: With publication

Additional Information
Who can access the data: researchers whose proposed use of the data has been approved
Types of analyses: for a specified purpose
Mechanisms of data availability: after approval of a proposal
